# Supplementary material for: A set of multi-entry identification keys to African frugivorous flies (Diptera, Tephritidae)
Source: Zookeys. 2014 Jul 24;(428):97–108. doi: 10.3897/zookeys.428.7366 (PMC4143993; doi:10.3897/zookeys.428.7366)
Supplement: Supplementary material 4 — Key to Capparimyia [file zookeys-428-097-s004.zip › SF4_ZooKeys_key to Capparimyia/lucid_player/help/help/whgdata/whnvl32.htm]

Search Letters


**#**
**A**
**B**
**C**
**D**
**E**
**F**
**G**
**H**
**I**
**J**
**K**
**L**
**M**
**N**
**O**
**P**
**Q**
**R**
**S**
**T**
**U**
**V**
**W**
**X**
**Y**
Z
